# Sleep profile status based on substance use, lipids and demographic variables in Tabari cohort study

**DOI:** 10.1016/j.sleepx.2022.100048

**Published:** 2022-05-04

**Authors:** Athena Enderami, Mahdi Afshari, Motahareh Kheradmand, Reza Alizadeh-Navaei, Seyed Hamzeh Hosseini, Mahmood Moosazadeh

**Affiliations:** a Psychiatry and Behavioral Sciences Research Center, Addiction Institute, Mazandaran University of Medical Sciences, Sari, Iran; b Pediatric Gastroenterology and Hepatology Research Center, Zabol University of Medical Sciences, Zabol, Iran; c Health Sciences Research Center, Addiction Institute, Mazandaran University of Medical Sciences, Sari, Iran; d Gastrointestinal Cancer Research Center, Non-communicable Diseases Institute, Mazandaran University of Medical Sciences, Sari, Iran

**Keywords:** Tabari cohort, Sleep profile, Substance, Lipid profile

## Abstract

**Background:**

This study aims to investigate the situation of sleep profile and its related factors in the Tabari Cohort Tabari (TCS) population.

**Methods:**

The information of 10255 of the Tabari cohort population in the enrolment phase was used in this study. The sleep profile data was collected and recorded by trained questioners. The sleep duration in day & night, the time interval between going bed and falling asleep, continuous use of sedatives, involuntary nap, limb hypermobility during sleep and shift working were determined for each person. Data analysis was performed by independent T test and Pearson correlation coefficient.

**Results:**

Mean, standard deviation, median, minimum and maximum of sleep duration in this population were 7.6, 1.6, 7.5, 0.5 and 17 h. Frequency of sleeping less than 6 h, 6–10 h and more than 10 h were 1168(11.4%), 8463(82.5%) and 624(6.1%) respectively. Prevalence of sleeping more than 10 h among men and women were 5% and 6.8% respectively (P < 0.001). Prevalence of sedative routine use among men and women were 4.7% and 9.6% respectively (P < 0.001). There were significant relationships between sleep duration and area residence, age group (P < 0.001), education level (P < 0.001), socioeconomic level (P < 0.001), triglyceride (P = 0.002), HDL-cholesterol (P = 0.013) and Cholesterol total (P = 0.021). There was a negative correlation between age and sleep duration (r = −0.062, P < 0.001).

**Conclusion:**

The results showed the association of the quality and quantity of sleep with personal, social, environmental and biological factors such as gender, age, economic status, educational status, and lipid profile. Therefore without proper intervention, the incidence of outcomes associated with these risk factors can be predicted in TCS In later years.

## Highlights

• Frequency of the sleep durations less than 6 h, between 6 and 10 h and more than 10 h among 10255 individuals in TCS were 11.4%, 82.5% and 6.1% respectively.
• The present study showed that several individual and social factors are responsible for quality and quantity of the sleep.
• Low sleep duration among subjects with total cholesterol less than 200, between 200 and 239 and more than 240 were 10.7%, 12% and 14.1% respectively.

## 1 Background

Sleep is a behavior, process and brain status with complex relationships with each other in different times and spaces [1,2]. Studies have showed an association between general health and 7–8 h of sleep [3]. Sleeping less than 7 h per day or low quality sleeping can lead to several disorders such as diabetes mellitus and insulin resistance, obesity, hypertension, cardiovascular problems and increased risk of death [[4], [5], [6]].

Patients with sleep disorders are classified to: those cannot sleep, those would not like sleep, those have excessive drowsiness during day and those with restlessness during sleep [7]. Age and gender are factors associated with such disorders which are more common among women as well as in the elderly. Women are complaining of difficulty in falling asleep, while, men are often suffer from light sleep, persistent sleep and respiratory disorders during sleep [8].

Sleep profile consists of two main aspects, sleep duration and sleep quality [9]. Sleep profile in various population has been investigated in previous studies mostly in diseased population [10,11].Few studies have been carried out regarding the quality and quantity of the sleep and its association with personal, social and biological factors among general population [12]. In present study data of more than 10000 participants, collected in enrollment phase of Tabari cohort has been utilized. The participants were enrolled from urban and mountainous regions of Sari district (North of Iran). These two populations differ significantly regarding epidemiological and life style related risk factors; [13]. Since the sleep pattern can play a main role in the daily function, the aim of present study was to investigate the sleep profile and its associated factors among the Tabari cohort population.

## 2 Methods

Data collected in the enrolment phase of the Tabari cohort was used in the current study. This study was approved by Mazandaran University of Medical science ethical committee (IR.MAZUMS. REC.1395.2524). Written informed consent form was obtained from all participants based on Declaration of Helsinki.

Tabari cohort is a part of the national cohort named as Prospective Epidemiological Research Studies in Iran (PERSIAN) [14,15]. More details of Tabari cohort has been explained in the cohort profile paper [13]. In the enrolment phase of the Tabari cohort, 10255 individuals aged 35–70 years old (7012 urban residents and 3243 rural residents) were recruited from Sari district (capital of Mazandaran province-North of Ian) using census method. The information of the Tabari cohort was collected using relevant questionnaire and blood sampling. Details of the standard questionnaire applied in the cohort has been explained elsewhere [[13], [14], [15]].

Some of the main variables measured in the cohort were as following:

Anthropometric factors including weight, height, waist circumference and hip circumference which were measured by trained staff according to a standard protocol. Height was measured by SECA 226 (SECA, Hamburg, Germany) meter. The weight was also measured using SECA 775 scale (SECA, Hamburg, Germany).

Blood samples were collected following 12 h fasting. For each participant 25 ml blood was collected. A portion of collected blood was used for biochemistry tests using BT 1500 (Biotechnical, Italy) and a complete blood count by Nihon (alpha cell counter Kohden, Tokyo, Japan). The rest of collected blood was separated into whole blood, plasma, serum and buffy coat and stored in freezers.

Sleep profile questions were questions regarding the time of sleep beginning (hour, minute), duration between bed time and sleeping time (minutes), time of waking up in the morning, time in which the subjects would like to be waken up (hour, minute), the time in which the individuals sleep during each day, working in the night hours during the last year (9 p.m.- 6 a.m.), limb hyper motility during sleep, napping during days and using sedatives more than two times per week. Finally, sleep duration was calculated and classified as less than 6 h, 6–10 h and more than 10 h. It should be noted that various definitions of these indices has been reported in the previous evidences. However, in the present study, it was define based on the Cappuccino et al. study and the expert opinion [16]. Moreover, the time duration between going to bed and sleep beginning was classified into less than 15 min, 16–30 min, 31–60 min and more than 60 min. The interview was conducted by two trained interviewers. One person also supervised the questioning process as a supervisor.

### 2.1 Statistical analysis

Data were analyzed using SPSS version 24 software. Data description was performed as percent frequency, mean, standard deviation, minimum, maximum and interquartile range. Comparing the time durations between demographic, behavioral and chemical factors was performed using Chi square test. Mean sleep duration was compared between different regions by independent T test. The correlation between sleep duration and age was investigated using Pearson correlation coefficient test. It should be noted that Bonferoni correction was carried out for multiple comparison and there was not any difference was between categories. P value less than 0.05 was considered statistically significant.

## 3 Results

Sleep profile of 10255 individuals in the Tabari cohort was investigated. Mean, median, minimum and maximum of sleep duration of study population were 7.6 ± 1.6, 7.5, 0.5 and 17 h respectively. Mean of sleep duration among urban and mountainous areas was 7.76 ± 1.59 and 7.23 ± 1.71 respectively (p < 0.001). Table 1 includes the participants’ characteristics based on residential area and, demographic, socioeconomic and behavioral factors.

**Table 1:** Characteristics of the participants in urban and rural areas in Tabari Cohort study population.

Variables		Urban; n(%)	Mountainous; n(%)	P-value
Gender	Male	2946(42)	1203(37.1)	<0.001
	Female	4066(58)	2040(62.9)	
Age group	35–44	2504(35.7)	827(25.5)	<0.001
	45–54	2488(35.5)	891(27.5)	
	55–70	2020(28.8)	1525(47)	
BMI	<25	1375(19.6)	1098(33.9)	<0.001
	25–29	3094(44.1)	1249(38.5)	
	≥30	2543(36.3)	896(27.6)	
Marital Status	Single/Widow/Divorce	507(7.2)	328(10.1)	<0.001
	Marriage	6505(92.8)	2915(89.9)	
**Education level**	No schooling	335(4.8)	1197(36.9)	<0.001
	1–5 years in school	1223(17.4)	1109(34.2)	
	6–8 years in school	832(11.9)	289(8.9)	
	9–12 years in school	2413(34.4)	483(14.9)	
	University/College	2209(31.5)	165(5.1)	
**Scio economic level**	1 (lowest)	408(5.8)	1643(50.7)	<0.001
	2	1149(16.4)	903(27.8)	
	3	1673(23.9)	377(11.6)	
	4	1807(25.8)	244(7.5)	
	5 (highest)	1975(28.2)	76(2.3)	
**Smoking daily**	Yes	640(9.1)	289(8.9)	0.723
	No	6372(90.9)	2954(91.1)	
**Hookah**	Yes	480(6.8)	103(3.2)	<0.001
	No	6532(93.2)	3140(96.8)	
**Drug use**	Yes	358(5.1)	266(8.2)	<0.001
	No	6654(94.9)	2977(91.8)	
**Alcohol**	Yes	763(10.9)	43(1.3)	<0.001
	No	6249(89.1)	3200(98.7)	

There was an inverse correlation between age and sleep duration (r = −0.062, P < 0.001). Of the cohort subjects, 25% were sleeping less than 6.5 h, 50% sleeping less than 7.5 h, 75% sleeping less than 8.5 h and 25% sleeping more than 8.5 h. In addition, frequency of the sleep durations less than 6 h, between 6 and 10 h and more than 10 h were 11.4%, 82.5% and 6.1% respectively. Frequency of sleep duration less than 6 h, 6–10 h and more than 10 h per 24 h among 3242 of individuals who live in mountainous region were 16.4%, 80% and 3.5% respectively. Corresponding rates for 7012 urban population were 9.1%, 83.7% and 7.3% respectively.The time interval between going to bed and beginning of sleeping was less than 15 min in 56.2% and more than 60 min in 7.4% of the cohort population. The time interval longer than 60 min among men and women was 3% and 10.4% respectively (p < 0.001). Consumption of sedative drugs more than two times per week was reported by 785(7.7%) of the cohort population. The situation of the other components of the sleep profile has been illustrated in Table 2.

**Table 2:** Sleep profile status in Tabari cohort population based on residence area and gender.

Variables		Total			Urban			Mountainous		
		Male	Female	P-value∗	Male	Female	P-value	Male	Female	P-value∗
Sleep status; hours	<6	389 (9.4)	779 (12.8)	<0.001	242 (8.2)	394 (9.7)	<0.001	147 (12.2)	385 (18.9)	<0.001
	6–10	3552 (85.6)	4911 (80.4)		2534 (86.0)	3333 (82.0)		1018 (84.6)	1578 (77.4)	
	>10	208 (5.0)	416 (6.8)		170 (5.8)	339 (8.3)		38 (3.2)	77 (3.8)	
**Duration Between to go to bed and sleep; minutes**	≤ 15	2796 (67.4)	2965 (48.6)	<0.001	2020 (68.6)	1921 (47.2)	<0.001	776 (64.5)	1044 (51.2)	<0.001
	16–30	857 (20.7)	1421 (23.3)		640 (21.7)	1055 (25.9)		217 (18.0)	366 (17.9)	
	31–60	373 (9.0)	1083 (17.7)		222 (7.5)	704 (17.3)		151 (12.6)	379 (18.6)	
	>60	123 (3.0)	637 (10.4)		64 (2.2)	386 (9.5)		59 (4.9)	251 (12. 3)	
**sedative routine use**	Yes	196 (4.7)	589 (9.6)	<0.001	149 (5.1)	403 (9.9)	<0.001	47 (3.9)	186 (9.1)	<0.001
	No	3953 (95.3)	5517 (90.4)		2797 (94.9)	3663 (90.1)		1156 (96.1)	1854 (90.9)	
**Involuntary nap**	Yes	840 (20.2)	1253 (20.5)	0.735	460 (15.6)	563 (13.8)	0.038	380 (31.6)	690 (33.8)	0.191
	No	3309 (79.8)	4853 (79.5)		2486 (84.4)	3503 (86.2)		823 (68.4)	1350 (66.2)	
**Moving a lot of legs during sleep**	Yes	443 (10.7)	824 (13.5)	<0.001	265 (9.0)	415 (10.2)	0.233	178 (14.8)	409 (20.0)	<0.001
	No	3538 (85.3)	4979 (81.5)		2597 (88.2)	3533 (86.9)		941 (78.2)	1446 (70.9)	
	I do not know	168 (4.0)	303 (5.0)		84 (2.9)	118 (2.9)		84 (7.0)	185 (9.1)	
**Night Working Shift**	Yes	631 (15.2)	143 (2.3)	<0.001	330 (11.2)	121 (3.0)	<0.001	301 (25.0)	22 (1.1)	<0.001
	No	3518 (84.8)	5963 (97.7)		2616 (88.8)	3945 (97.0)		902 (75.0)	2018 (98.9)	
**Has day time sleep**	Yes	2378 (57.3)	3170 (51.9)	<0.001	1801 (61.1)	2326 (57.2)	0.001	577 (48.0)	844 (41.4)	<0.001
	No	1771 (42.7)	2936 (48.1)		1145 (38.9)	1740 (42.8)		626 (52.0)	1196 (58.6)	

∗: P-value based on the results of Chi-squared test.

Table 3 compares the sleep profile of the cohort subjects based on demographic, behavioral and laboratory factors. Frequency of sleeping shorter than 6 h in subjects aged 35–44, 45–54 and 55–70 years were 8.4%, 11.1% and 14.5% respectively (p < 0.001). There were no significant relationships between sleep duration and BMI (p = 0.128), marital status (p = 0.351), cigarette smoking (p = 0.371), opium addiction (p = 0.594), hookah (p = 0.051), alcohol consumption (p = 0.096). Frequency of low sleep duration (shorter than 6 h) among subjects with different socioeconomic levels were 15.9%, 14.2%, 10.3%, 9.1% and 7.4% respectively for the first, second, third, fourth and fifth levels of socioeconomic status (p < 0.001). Low sleep duration among subjects with total cholesterol less than 200, between 200 and 239 and more than 240 were 10.7%, 12% and 14.1% respectively (p = 0.021). The time interval between bed time and beginning the sleep based on subjects with different demographic, behavioral and laboratory factors has been illustrated in Table 4.

**Table 3:** Sleep status of Tabari cohort population based on different factors.

Variable		<6 h	6–10 h	>10 h	P-value∗
Area residence	Urban	636(9.1)	5867(83.7)	509(7.3)	<0.001
	Mountainous	532(16.4)	2596(80.0)	115(3.5)	
**Age group**	35–44	280(8.4)	2842(85.3)	209(6.3)	<0.001
	45–54	375(11.1)	2813(83.2)	191(5.7)	
	55–70	513(14.5)	2808(79.2)	224(6.3)	
**BMI**	<25	287(11.6)	2057(83.2)	129(5.2)	0.128
	25–29	478(11.0)	3602(82.9)	263(6.1)	
	≥30	403(11.7)	2804(81.5)	232(6.7)	
**Education level**	No schooling	291(19.0)	1172(76.5)	69(4.5)	<0.001
	1–5 years in school	325(13.9)	1835(78.7)	172(7.4)	
	6–8 years in school	119(10.6)	924(82.4)	78(7.0)	
	9–12 years in school	251(8.7)	2465(85.1)	180(6.2)	
	University/College	182(7.7)	2067(87.1)	125(5.3)	
**Marital status**	single-widow-divorce	106(12.7)	674(80.7)	55(6.6)	0.351
	marriage	1062(11.3)	7789(82.7)	569(6.0)	
**Scio economic level**	1 (lowest	326(15.9)	1642(80.1)	83(4.0)	<0.001
	2	292(14.2)	1626(79.2)	134(6.5)	
	3	212(10.3)	1691(82.5)	147(7.2)	
	4	186(9.1)	1721(83.9)	144(7.0)	
	5 (highest)	152(7.4)	1783(86.9)	116(5.7)	
**Smoking daily**	Yes	104(11.2)	778(83.7)	47(5.1)	0.371
	No	1064(11.4)	7685(82.4)	577(6.2)	
**Hookah**	Yes	56(9.6)	502(86.1)	25(4.3)	0.051
	No	1112(11.5)	7961(82.3)	599(6.2)	
**Drug use**	Yes	64(10.3)	519(83.2)	41(6.6)	0.594
	No	1104(11.5)	7944(82.5)	583(6.1)	
**Alcohol**	Yes	75(9.3)	687(85.2)	44(5.5)	0.096
	No	1093(11.6)	7776(82.3)	580(6.1)	
**TG**	<150	712(11.9)	4944(82.6)	332(5.5)	0.002
	150–199	226(11.4)	1636(82.7)	117(5.9)	
	≥200	230(10.1)	1883(82.3)	175(7.6)	
**HDL-C**^a^	>40 for male or >50 for female	793(11.7)	5625(82.7)	382(5.6)	0.013
	≤40 for male or≤50 for female	375(10.9)	2838(82.1)	242(7.0)	
**Cholesterol total**	<200	701(10.7)	5444(83.2)	401(6.1)	0.021
	200–239	327(12.0)	2222(81.8)	167(6.1)	
	≥240	140(14.1)	797(80.3)	56(5.6)	

∗: P-value based on the results of Chi-squared test.

a **HDL-C: HDL-cholesterol**.

**Table 4:** Time interval between bed time and sleep time among Tabari cohort population based on different variables.

Variable		≤15 min	16–30 min	31–60 min	>60 min	P-value∗
Area residence	Urban	3941(56.2)	1695(24.2)	926(13.2)	450(6.4)	<0.001
	Mountainous	1820(56.1)	583(18.0)	530(16.3)	310(9.6)	
**Age group**	35–44	1981(59.5)	813(24.4)	386(11.6)	151(4.5)	<0.001
	45–54	1868(55.3)	762(22.6)	496(14.7)	253(7.5)	
	55–70	1912(53.9)	7.3(19.8)	574(16.2)	356(10.0)	
**BMI**	<25	1485(60.0)	517(20.9)	319(12.9)	152(6.1)	<0.001
	25–29	2469(56.9)	1001(23.0)	577(13.3)	296(6.8)	
	≥30	1807(52.5)	760(22.1)	560(16.3)	312(9.1)	
**Education level**	No schooling	783(51.1)	221(14.4)	296(19.3)	232(15.1)	<0.001
	1–5 years in school	1183(50.7)	528(22.6)	383(16.4)	238(10.2)	
	6–8 years in school	583(52.0)	300(26.8)	166(14.8)	72(6.4)	
	9–12 years in school	1625(56.1)	723(25.0)	389(13.4)	159(5.5)	
	University/College	1587(66.8)	506(21.3)	222(9.4)	59(2.5)	
**Marital status**	single-widow-divorce	389(46.6)	183(21.9)	159(19.0)	104(12.5)	<0.001
	marriage	5372(57.0)	2095(22.2)	1297(13.8)	656(7.0)	
**Scio economic level**	1 (lowest	1092(53.2)	361(17.6)	377(18.4)	221(10.8)	<0.001
	2	1032(50.3)	499(24.3)	308(15.0)	213(10.4)	
	3	1113(55.3)	504(24.6)	281(13.7)	132(6.4)	
	4	1208(58.9)	488(23.8)	261(12.7)	94(4.6)	
	5 (highest)	1296(63.2)	426(20.8)	229(11.2)	100(4.9)	
**Smoking daily**	Yes	543((58.4)	215(23.1)	116(12.5)	55(5.9)	0.089
	No	5218(56.0)	2063(22.1)	1340(14.4)	705(7.6)	
**Hookah**	Yes	382(65.5)	125(21.4)	56(9.6)	20(3.4)	<0.001
	No	5379(55.6)	2153(22.3)	1400(14.5)	740(7.7)	
**Drug use**	Yes	365(58.5)	134(21.5)	89(14.3)	36(5.8)	0.359
	No	5396(56.0)	2144(22.3)	1367(14.2)	724(7.5)	
**Alcohol**	Yes	533(66.1)	174(21.6)	73(9.1)	26(3.2)	<0.001
	No	5228(55.3)	2104(22.3)	1383(14.6)	734(7.8)	
**TG**	<150	3408(56.9)	1330(22.2)	832(13.9)	418(7.0)	0.066
	150–199	1079(54.5)	421(21.3)	310(15.7)	169(8.5)	
	≥200	1274(55.7)	527(23.0)	314(13.7)	173(7.6)	
**HDL-C**	>40 for male or >50 for female	3899(57.3)	1463(21.5)	957(14.1)	481(7.1)	0.006
	≤40 for male or≤50 for female	1862(53.9)	815(23.6)	499(14.4)	279(8.1)	
**Cholesterol total**	<200	3771(57.6)	1456(22.2)	870(13.3)	449(6.9)	<0.001
	200–239	1466(54.0)	613(22.6)	420(15.5)	217(8.0)	
	≥240	524(52.8)	209(21.0)	166(16.7)	94(9.5)	

∗: P-value based on the results of Chi-squared test.

## 4 Discussion

This study investigates the sleep profile of 10255 Mazandaran residents in northern part of Iran (Tabari cohort population). In the present study, the frequency of sleeping less than 6 h/longer than 10 h among urban residents, mountainous residents, men and women was estimated as of 9.1%/7.3%, 16.4%/3.5%, 9.4%/5% and 12.8%/6.8% respectively. In addition, frequency of different sleep profiles was the same among those with and without smoking habit, opium addiction, alcohol consumption and those with different body mass indices. However, significant differences were observed between sleep profiles of the participants with various lipid profiles, educational status, age groups and socioeconomic levels.

In the present study, the prevalence of sleep less than 6 h and more than 10 h in the mountainous area was higher in urban areas. This finding is consistent with results of Xiang et al. conducted among 5926 general population [17]. Another study performed in China, showed prevalence of insomnia among rural residents was significantly higher than urban residents (29.4% vs 25.5% respectively) [18].

Finding of present study showed, average time of sleep was 7.76 ± 1.59 and 7.23 ± 1.71 h in urban and mountainous areas respectively. More than two-third of them had less than 7 h sleep per 24 h. Such amount of sleeping is in keeping with those reported from the other communities [19,20]. Considering the recommendations for enough sleeping for general health [3], such sleep pattern in this population can lead to different disorders in long term. Therefore, it is necessary to plan suitable strategies for improvement of personal behavior such as regular sleep pattern, specifying the exact time for going bed in the night and waking up in the morning, designing a quiet environment, avoiding from electronic devices and lavish meals, alcohol and caffeine.

The current study showed higher rates of sleep duration less than 6 h and more than 10 h among women and also a negative association between ages and sleep duration. Evidences show increasing the amount of complaints of sleep problems with ageing especially among women [20,21].

The time duration between bed time and sleeping time was shorter than 15 min in more than half of the Tabari population and just 7.4% of them had sleeping time interval longer than 60 min. Exelmans et al. showed such interval shorter than 15 min in one-fifth of the Belgian adults, while, 9.5% of them needed more than 1 h to sleep [22]. Such difference might be partially due to the different urban/rural composition of the Tabari cohort population and the populations investigated in the other studies. However, no appropriate evidences were found regarding the relationship between this index and the residence area. Out of our population, 7.7% reported using sedative drugs at least two times per week which was similar to those reported from Brazil. (7.6%) [23].

Involuntary naps in the present study was 20% which was in agreement with that reported by Ohayon et al. [24]. However, Van der Spuy et al. reported lower rates and also introduced several determinant factors such as respiratory co-morbidities, conditions of poverty, and loud snoring [25].

The current study showed that about 10% of men and 13% of women had limb movements during sleep which was similar to those found by Kerkhof GA et al. [20] but was higher than the prevalence rates of the other studies [26,27]. It should be noted that most of people with restless leg syndrome are suffering from such adverse experiences including rhythmic and periodic movements of legs during sleep. These symptoms are often revealed by the patients’ roommates [7].

In the Tabari cohort population, night work was considerably higher among men than women. This characteristic can be considered as a medical problem so that 32.1% of people with shift work and 26.1% of those with shift work switching between days and nights had reported minimum criteria of sleep disorder. These shift work switching has been investigated from different aspects indicating changes in the circadian rhythm of the persons leading to sleep and mental disorders as well as different work errors and high economic costs in long term [28,29].

Our results showed that approximately half of the Tabari cohort population had day time sleeping which was much longer than the times reported for the most other communities in the previous literature [30,31]. Although we did not find any relationship between short sleeping and BMI, previous evidences showed negative correlations between BMI and sleep duration so that those with shorter sleep durations have higher risk of developing obesity [32], [33]. A study carried out among US population aged over 40, mean of BMI in patients with insomnia (32.9%) was significantly higher than those without insomnia (28.5%) [34].

We found negative association between the educational level and short sleeping. Our results was in agreement with those in the Netherland and United States [20].

Although we did not find any relationship between sleep duration and tobacco or alcohol use, the results showed that those having these habits fall in asleep much faster than the other individuals. Previous studies indicated that tobacco smoking or secondary exposure to its smoke and also caffeine and alcohol consumption lead to onset of different sleep disorders [35,36]. Current cigarette smoking and current alcohol drinking caused significantly 47% and 26% higher odds of developing insomnia in general population [18]. Khazaei et al. showed that opium dependent patients without treatment had higher prevalence of sleep disorder compared to those receiving methadone maintenance therapy [37].

In this study short sleep duration had negative correlation with the socioeconomic status. These finding are in parallel with the previous literatures revealed that the highest complaints of sleep disorders are among the low socioeconomic groups [38,39]. The exact mechanism for the relationship between sleep status and socioeconomic level has not yet been specified. However, some probable reasons might be involved. For example, socioeconomic situation can play as an intermediate factor affecting the sleep status through induction of stress, anxiety and other psychological problems. On the other hand, socioeconomic situation associates with the level of welfare and access to living facilities such as convenient environment for sleeping and rest. Socioeconomic status effects on working hours and hard work.

The association between lipid profile and sleep profile showed longer sleep duration among those with higher triglyceride and lower HDL-C. Many studies carried out in different parts of the world have shown the relationship between abnormal lipid profile and low quality sleeping [[40], [41], [42], [43]].

One of the limitations of the present study was that data regarding sleep quality indicators are subjective and were collected based on self-reporting which can be different from the results of the objective measures. However, it seems that such error is negligible because of the large samples size. It should be noted that the present study aimed to describe the sleep profile of the Tabari cohort population. Therefore causal relationship is not the main focus of the study and multivariate analyses were not used in the study.

## 5 Conclusion

The results showed the association of the quality and quantity of sleep with personal, social, environmental and biological factors such as gender, age, economic status, educational status, and lipid profile. Since the lack of enough sleep can lead to cognitive, mental and mood disorders, fatigue and weakness, disturbances in physical activities, poor decision making, physiological and hormonal disorders, it is necessary to increase the public knowledge about the importance of the sleep to prevent onset of such consequences.

## Ethics approval and consent to participate

TCS was approved by Mazandaran University of Medical science ethical committee (IR.MAZUMS. REC.1395.2524). Written informed consent form was obtained from all participants.

## Funding

This study was conducted with a financial support of the 10.13039/501100003969Ministry of Health and Medical Education (grant No. 700/534) and 10.13039/501100004160Mazandaran University of Medical Sciences (grant No. 2524).

## Authors’ contributions

MM, MK, AE and MA acquired data, performed the statistical analyses, interpreted data, and drafted and revised the manuscript for important intellectual content and approved the final version. RA and SHH interpreted data, reviewed the analyses and approved the final version.

## Consent for publication

Not application.

## Availability of data and materials

The datasets used and/or analyzed during the current study are available from the corresponding author on reasonable request.

## Declaration of competing interest

The authors declare that they have no Competing interests.
